# How Is Quality of Care in Home Healthcare Created? A Qualitative Study of Health Professionals’ Perspectives

**DOI:** 10.3390/healthcare10061021

**Published:** 2022-05-31

**Authors:** Sigrid Nakrem, Katrine Kvanneid

**Affiliations:** 1 Department of Public Health and Nursing, Faculty of Medicine and Health Sciences, Norwegian University of Science and Technology (NTNU), 7491 Trondheim, Norway; katrine@kvanneid.com; 2 DPS Solvang, Sørlandet Hospital, Sørlandet Sykehus HF (SSHF), 4604 Kristiansand, Norway

**Keywords:** home healthcare, patient safety, qualitative method, quality of care, nursing

## Abstract

The demographic challenges with an increase in older adults in need of nursing care has put home healthcare services under pressure. However, research on what constitutes quality of home healthcare services and what factors influence good nursing care and patient safety is scarce. The aim of this study was to gain insight into health professionals’ perceptions of how quality of care in home healthcare is created and what factors put patient safety at risk. The present study was a qualitative study with semi-structured interviews of eight health professionals working in home healthcare services. Qualitative content analysis was used. Four categories of factors the staff thought had to be present to provide good quality services were identified: (1) A workplace with adequate competence; (2) Communication, information flow and collaboration; (3) Continuity and organisation of care; and (4) Resources. Conclusions: The healthcare professionals perceived that the quality of the services overall was good, and if important factors were present, quality of care was achieved. However, they pointed out some factors that were important to prevent inadequate care and improve services, as quality of care was at risk when deficiencies in these areas occurred.

## 1. Introduction

Home healthcare is a relatively novel service in many countries and exists in various forms worldwide. In some countries only practical assistance in the home is offered, while others have services that include advanced medical nursing care such as hospital-at-home. The way it is organized also varies, for instance, some countries have only private services, while others provide a fully financed public service [1]. Home healthcare service is the largest primary healthcare sector in Norway, and the sector is predicted to increase because of de-institutionalization and the increase in home-dwelling patients in all age groups in need of advanced nursing care [2,3,4]. In Norway, the service has evolved from assistance in households, e.g., child care when a parent had become sick, to an ever more complex care [5]. In 2017, more than 190,000 patients received municipal healthcare, encompassing approximately 143,000 man-labour years. This became a statutory obligation in primary healthcare in 1984 [6]. A new statute for municipal healthcare from 2012 [7] and the coordination reform [8] underpinned the municipalities’ responsibility to provide nursing services at home. In addition, home healthcare was divided into nursing/healthcare and practical assistance in the household, and became more specialized, e.g., rehabilitation services, dementia care, and palliative care. These changes—together with implementation of contemporary care philosophies where more people (especially older adults with chronic diseases) are expected (and want), to live at home longer—has put home healthcare under pressure [3,9,10].

The municipalities in Norway can organize home healthcare independently, and there are few guidelines on the provision of care. Most municipalities organize the home healthcare according to a model for New Public Management where purchaser (allocation office) and provider (staff) are split into different units within the municipality [11,12]. The provision of care is delivered under the “National Regulation of Quality of Care” [13]. This regulation aims to ensure that residents’ basic needs are met, including their psychological and physical needs, and that their dignity, autonomy, and self-respect are preserved. Healthcare professionals’ individual responsibility to deliver care that is safe and of high quality is regulated by the “National Health Personnel Act” [14], as well as the professional code of ethics [15]. The Norwegian national regulation of management and quality improvement in healthcare services obliges the municipalities to monitor the overall quality and safety [16].

Quality of care is defined as the “degree to which a set of inherent characteristics fulfils requirements” [17] (p. 24). More specifically for healthcare, the Institute of Medicine provided an internationally recognized definition: “Quality of care is the degree to which health services for individuals and populations increase the likelihood of desired health outcomes and are consistent with current professional knowledge” [18] (p. 21), and further outlined the six domains of quality of care: safety; effectiveness; patient-centeredness; timeliness; efficiency; and equity [19] (pp. 39–40). This definition can be accompanied by the concept of patient safety, where the aim is to prevent and reduce risks, errors, and harm to patients during the provision of health care [20]. Quality of care encompasses a compound of properties on a continuum between low quality, characterized by frequently missed care that harms patients, and high quality, where patient safety is fundamental and services are characterized with high patient satisfaction [21]. Patient satisfaction is often the outcome that is measured when investigating quality of care. A study by From et al. [22] found that home healthcare patients emphasized factors of being respected as an individual, engagement and compassion of nurses, keeping their daily routines, continuity, and trust in the services. In a Norwegian study, the patients were generally satisfied with the quality, but missed more time for longer conversations with the nurses [23]. Continuity in care is important for patient satisfaction [24]. However, it was found that during a period of four weeks the same nurse would visit the same patient only three times [25]. Furthermore, the competence level of nurses in Norwegian primary care was found to be unsatisfactory, particularly in advanced care [26,27,28]. International research has pointed out that a diversity of factors related to leadership, stress and work climate, organization of work, staffing, time resources and patient-centred care influence quality of care from the perspective of healthcare professionals [29,30,31,32,33,34].

It is indicated that there is a need for improvements in Norwegian home healthcare [4,24], while research that can guide municipalities on how to monitor quality and safety in home healthcare is scarce. There is a need for better understanding of what influences quality improvement in home healthcare and how to mitigate patient safety risks. The aim of the study was therefore to explore how quality in home healthcare is created. More specifically, we wanted to investigate the prerequisites of creating high quality care in home healthcare, and what creates the opposite, along with what factors put home healthcare quality and safety at risk for patients receiving advanced nursing care at home.

## 2. Materials and Methods

### 2.1. Design

A qualitative design was applied to achieve an in-depth understanding of quality of care and patient safety in advanced home healthcare, from professional healthcare workers’ perspectives [35,36]. The study is connected to a larger research project on elder abuse and neglect financed by the Research Council of Norway (ref. nr 262697).

### 2.2. Recruitment and Sample

The participants were recruited by contacting the head of home healthcare in six municipalities. One urban municipality and one rural municipality agreed to participate, and information was distributed to potential participants by the head. In addition, one participant was recruited by contacting a home healthcare unit in a larger urban municipality. Inclusion criteria was being a Registered Nurse (RN) or Licensed Practical Nurse (LPN) in at least a half-time position and having worked in home healthcare for more than one year. Five RNs and three LPNs participated in the study. 

### 2.3. Data Collection

A semi-structured interview guide based on literature and the professional experience of the authors was developed and used to guide the interviews (see Table 1). The interview guide was piloted in one interview (data not included in the study) and adjusted according to feedback from this interview. The interviews were conducted by the second author of this paper between October 2019 and February 2020. Individual interviews took place in a separate room at the participants’ workplaces, except for one that was conducted in a place away from the participant’s workplace. The interviews lasted 12–50 min (median time 27.8 min), and were audio recorded and transcribed verbatim by the second author immediately after each interview.

### 2.4. Data Analysis

Graneheim and Lundman’s manifest and latent content analysis was used to analyse the data. The method is inspired by Giorgi’s four steps in the phenomenological approach to the analysis [37]. The interviews were first read in their entirety to get an overview. Next, units with an independent meaning were identified and further condensed. The following step was to abstract the meaning and subsequently code the meaning units. Finally, an interpretation of the underlying meaning was categorized, and thereby the description moved from manifest to latent content of the text. In this part of the process, the tool NVivo (QSR International^®^, Chiyoda City, Japan) was used and provided a structure for categorizing the data. To ensure that the analysis was performed reliably, the authors met during steps three and four for a critical review of preliminary codes, sub-themes, and themes, and discussed the analysis results to reach an agreement. The authors then selected quotes that provided a representative picture of the material. 

### 2.5. Ethical Considerations

Ethical approval for this study was given by the Norwegian Centre for Research Data (NSD), reg.no. 316178. All participants received oral and written information about the study prior to the interview and gave written consent to participate. All identifiable characteristics are excluded from the presentation of data to ensure the anonymity of all individuals.

## 3. Results

The five RNs and three LPNs were all female, worked in an 80–100% position and had worked for 2–22 years in home healthcare (median 6.25 years). The main finding was that their service unit all in all delivered care of high quality. Still, participants highlighted some factors that they perceived would affect quality of care and were essential to prevent the risk of deficiencies or low quality of care. Four categories emerged from the analyses (Table 2) and are presented below.

### 3.1. A Workplace with Adequate Competence

An important contributing factor was competence and experience among staff. Maintaining an adequate professional community over time was essential. Participants stated that a service with high quality consisted of units with competent and skilled nurses. The nature of advanced home healthcare services required highly skilled nurses, and if the professional nurses were replaced by less trained assistants it would result in missed care and the risk of deficiencies. The participants emphasized that the work in home healthcare requires more than just a desire to help people; more qualified staff such as specialized nurses was needed: “*We need people with competence, yeah. We can’t just like pick people up off the street and think that it’ll all work out. There needs to be quality in each step along the way” (participant 7).* Many patients were dependent on advanced medical technology, and skills and training to use these technologies were needed: “*So you need to be good at using the [assistive technology], really, and then you need to have both the knowledge and be conscious that it’s actually wise to use” (participant 7).* Staff mix and availability of multi-disciplinary professionals were also important since the patients had complex needs. Moreover, nurses that have less experience were considered by the participants as risk factors for deficiencies: “*And maybe there we are a bit weak since they [the new graduates] don’t have that experience, they might not have the same clinical judgment as those who have worked longer” (participant 1).*

Opportunities to continuously keep up to date and having academic progress in the field were preventive factors for deficiencies, and the participants wanted the management of the services to accommodate this in their workplace:

*You need to have somewhere [to go], where else can you get information about things that change? Diabetes is just one example, in that field there has been big changes the past years, and I don’t think there are many people who are aware of that (participant 1).*

Having access to continuing education and relevant training courses would increase their confidence in care procedures, which could promote patient safety, in particular for patients with advanced needs:

*You’re often just thrown right into something, and the thought is like “yeah, but you got this, no problem.” But the truth is, I will be able to do these things, but… I use a lot more time to learn and do these things if I don’t have training in it. Because then, I need to like figure it out all for myself, so when you can get just that little extra [training]… to know that I can do this, this I am confident with (participant 6).*

### 3.2. Communication, Information Flow and Collaboration

Participants highlighted throughout the interviews that quality of care was undisputedly linked to always trying to do the best for the patient in their care pathway. One way of securing this was the ability to see the patients as individuals, to meet their specific needs, respect their preferences, and perform care that was agreed upon in the contract:

*Well, we do have different views on what is important, and if you don’t communicate enough with the patient, and like don’t ask them what is important for them, then it is really easy to just do what you think is okay, but that might not be actually what they want (participant 6).*

Communicating well with the patient also included being polite and compassionate and not doing the tasks too hastily. Good communication and professional clinical gaze were important to be able to detect changes in the patient’s status, according to the participants.

Just as important to preventing errors was the communication between staff. Good communication and safe information flow prevented misunderstandings and missed care that could be devastating for the patient: “*For example, if there are some misunderstandings, the people that need help experience it as pretty unfortunate. There is basically a communication failure… we’ve got to be better at that” (participant 1)*. Missing notifications from a patient would also lower the trust in the services, and one participant stated that it was important for the experience of quality of care to keep promises and be on time. Good communication routines included written information rather than the oral transfer of messages. It was important to have an adequate system for reporting and documenting care: “*Things kind of fall between the cracks. There are many steps before the messages arrive to those who should get them, and along the way, they disappear” (participant 3).* Quality of care and patient safety was enhanced with good practice for information handling, even if the workdays were stressful and staff lacked time to transfer information between them during shifts. This included a structured electronic patient record where important information about the patient was updated and clearly written: “*Documentation is actually super important because if something happens, then there is an adverse event. In the worst case there can be a lawsuit from that type of thing, and in a way, documentation is our own evidence” (participant 4).*

Participants described how too little knowledge about the specific care tasks, or less possibility to prepare before visiting the patient if the information was poorly described, could lower the quality of care. Continuity in care could be jeopardized if it was unclear which assessments were done earlier by other nurses, and what nursing intervention had been tried before. Lack of information would lead to stress and discomfort, which again was a risk for errors, according to the participants:

*When you’re standing and looking at the clock and are unsure which order things should be done in, and unsure about how the patients like it, and unsure about pretty much everything… then the quality can go down a bit because things are easily forgotten, and you experience a high level of stress (participant 3).*

Collaboration and good interaction with key stakeholders and other professionals such as physicians, physiotherapists, and specialists in mental health and substance abuse was an essential factor to enhance quality of care. This required, however, that the multi-disciplinary support was timely and helpful:

*Doctors are better to answer e-links now, the hospital is better at following up what we send to them, and the other way around. But also physiotherapists, the drug addiction team, all of these... It is important with really close cooperation since we have so many different patients (participant 3).*

Participants highlighted that good collaboration with their leaders provided opportunities for developing autonomy in work at the same time as professional support was available when needed:

*Now that we have management that are a bit closer to us, and are there to help us remember things, that helps to quality assure the job we do. They’ve got our backs and are very welcoming when it comes to questions and take professional subject matter discussions with us (participant 1).*

A good relationship with the patient’s relatives was important in some cases to ensure quality of care, especially in difficult situations when nurses got good advice from relatives who know the patient better.

### 3.3. Continuity and Organization of Care

Participants indicated that a variety of factors related to how the home healthcare services were organized had an impact on quality of care and patient safety. The care was organized in work lists or lists of tasks based on nursing plans for the patients. However, the work lists were perceived as a challenge by the participants in their daily work, especially when it came to organizing care according to individual needs. Often the lists were based on geographical location and how care could be performed more effectively rather than the individual patient’s care plan:

*You could make the lists based on the right competence. Many people think about geography when they make the lists, I believe, first because that’s the quickest thing to do, because when there are two patients [who need help] in the same building, you know (participant 1).*

Participants expressed that this was an area for improvement, and the main issue was how to ensure appropriate competence to the right patients: “*That you assure that those who need a nurse’s help, get a nurse’s help. And that the temps who are hired in can warm up the dinner, you know” (participant 5).* Often the problem was that those who make the lists do not know the patients and their individual care needs. In other cases, the participants indicated that the principle was that all tasks should be equally distributed no matter what competence the employee possesses. This often led to RNs doing multiple tasks in addition to their nursing tasks, while assistants were less busy:

*It’s often that I could have been out on an assignment, for example follow someone to exercise class or fix lunch or something an assistant can also do. So, it is better that the assistants have something to do the hours that they are at work, so that I can do other things (participant 4).*

One participant said that despite this problem having been reported to the leader, and that they had suggested that work lists should be made by the RNs, nothing changed. Not being listened to, frustrated the participant. Because of the poor organization of tasks, the nurses had to spend their crucial time on reorganizing the lists daily to ensure adequate competence for each task on the lists.

Another consequence of the way their work was organized was the large number of different staff that the patient had to relate to, and this made it unsafe for many patients: *There will, you know, be a little more uncertainty and maybe a worse job will be done when there are many different [health professionals] that go [home to a patient] (participant 8).* The participants connected this challenge to stability in the staff group and good working relationships. A stable staff group with nurses in large positions ensured ownership and pride of their work: “*If you work in a full-time position then you have a better overview and you have a much larger sense of ownership to your job. And I actually think that maybe then you do a better job too” (participant 7).* Participants felt that it was easier to achieve good teamwork if the staff knew each other well and were trusting of each other: “*That you can trust that your colleague does a good job…so that we all make it work together” (participant 2).* Good collaboration in the staff group was important for adequate follow-up concerning patients’ needs. Participants in one municipality said that if a challenge arises in a patient, the nurses come together to solve it. The participants believed that being able to exchange experiences, collaborate with colleagues and have someone to talk to contributed to quality in the services.

### 3.4. Resources

The main resource factors that participants indicated had an impact on quality and safety were staffing and time. All participants expressed that staffing levels were too low considering the patients’ needs for care. They considered that more staff would give them more time with each patient and make the care more flexible and able to adjust to varying needs over time. Inadequate staffing was particularly problematic during evening shifts and weekends, since small unforeseen events could be detrimental to scheduled care plans and influenced the quality of direct nursing care: “*If you don’t have enough people at work then a worse job will be done… You might not be able to do everything you need to” (participant 8).* Hiring part-time staff or nurses from temporary staff contracting services was perceived as a poor solution, as these employees were less engaged in their job and did not have the information they needed to do a good job for the patients, especially if there were changes in the patients’ needs: “*It could be that they don’t feel that they have the attachment to the job in the sense that they might feel like ‘yeah, whatever, maybe it’s no problem” (participant 6).* Participants expressed that the time pressure forced them to make difficult prioritizations between tasks. Often this led to too little time to sit down and talk to patients which was important for the patients’ quality of life and mental health. It could also be difficult to get an overview of important care tasks, which made them feel less in control of the care service. Participants believed that it was important to reflect upon their work; however, there was seldom time for this: “*…reflect over that maybe I did something right, am I sure it’s okay when I leave?” (participant 2).* Reporting adverse events caused by lack of time and inadequate staffing was important with regard to being able to improve and possibly get more resources:

*Reporting adverse events are not meant to tattle on someone or that someone will hang out to dry in some way… [it] just points out the lack of resources, that there are too few staff at work for example… these types of things can be revealed if you are good at reporting adverse events (participant 7)*

## 4. Discussion

The main finding of the present study was that nurses in home healthcare generally perceived the quality and safety to be good. To improve quality and safety the main interventions were related to strengthening the clinical teams with adequate competence. Patient safety risks were present when information was lacking about the patients or there were challenges in the information flow. Furthermore, the participants suggested that the organization of care had to be improved to secure continuity of care and a good work climate. Having adequate resources regarding staffing and time was crucial in delivering safe care.

Despite the satisfaction with the competence of staff in the participants’ units, they were worried about the lack of training and the overall competence in the care team in the future. This was especially connected to the pressure of having to care for an increasing number of home-dwelling patients with complex needs. They expressed that a strong nursing team is important to secure high quality and safety. Previous research has shown that adequate skilled healthcare professionals are lacking in home healthcare [27]. The participants in our study felt that training and education contributed to being more confident in care tasks and were worried that assistants and temporary employees could pose a threat to patient safety. Other studies have found that nurses in home healthcare were dissatisfied with the ability to utilize their competence due to insufficient organization of tasks or that staff lacked the necessary competence [26,28,38]. This worry is also shared by relatives in another study where the informal caregivers stated that relevant competence of the healthcare professionals in managing the complexity of the patients’ health services is essential [39].

The participants provided details of how communication skills were crucial to increase quality and safety. Good communication could improve the information flow between patients, relatives, and the health professionals, and between different healthcare providers. The goal of healthcare is better outcomes for the patients, and good cooperation between all relevant stakeholders and actors is crucial for patients with complex needs who need multidisciplinary care [40]. Healthcare professionals, especially nurses in home healthcare, are in a position to be an advocate for the patient, provided they have adequate information about the individual needs of the patient and the context in which the patient experiences [41]. Having good knowledge of patients’ needs and performing care adjusted to the individual patient is the essence of person-centred care, resulting in quality of care [33]. This is also important for the nurses to be able to make the correct decisions for the patient [42]. However, often the health professionals experience limited time getting to know the patients and understanding what is important for each patient in their current situation [39]. Short visits and stressful working situations might put safety at risk because the patients might feel that it is not suitable to bring forward their worries and needs [43,44].

Staff stability and sense of ownership in their work was emphasized by participants as key factors for quality of care in the home healthcare organizations. The way the work was organized and how tasks were divided between the staff on a daily basis had tremendous impact on the work situation. In a meta-synthesis, the striking findings were that healthcare professionals struggle to balance patients’ needs and the demands of the organization for efficiency [41]. It has been found that the healthcare professionals’ working conditions are important for quality of care [34]. Various organizational factors have been found to inhibit person-centred care, and nurses report that the pressured working conditions change the focus to more task-oriented care with emphasis on medical aspects rather than the humanity of care [41,45]. On the other hand, for the patients with less complex needs, short and task-oriented visits provide patients with an assurance that they can continue to live at home, and this could give them a smooth introduction to receiving help from home healthcare services [46].

Participants expressed another important factor for quality and safety, which was the lack of continuity due to the large number of staff the patients had to relate to. They related this to organization of care, and the system for generating work task lists. Lack of continuity has an impact on how well patients are followed up with and hinders the professional nurses in detecting signs of deterioration in patients’ clinical status [2,25]. High quality of care in home healthcare means the “right help, by the right professional at the right time and right location” [2]. The participants in our study expressed the importance of having clear consciousness of what constitutes good quality of care, and to report adverse events. They emphasized good support from leaders, especially when patient safety was at risk. Being able to report adverse events without being judged was important for the participant, as they saw that it could improve quality and safety. Good follow-up on adverse events has been found to increase the learning from these events [47]. Working systematically with patient safety issues has also been found to increase openness about safety risks and to heighten perceived professionalism among nurses [48].

### Strengths and Limitations of the Study

The information-rich interviews with the nurses are the main strength of this study. Even though the sample was relatively small, we perceived that saturation was achieved already after six interviews, and that no more data was obtained in the last two interviews. This might be explained by the focused interview guide and that all nurses were working in similar organizations [37]. On the other hand, some of the participants might have been reluctant to criticize their own workplace and express that care quality was low, or to give notice of safety issues. This is particularly relevant since they were recruited to the study by their leaders. Our impression was, however, that the participants were honest in their statements, and had a genuine wish for communicating what was good care in their organization and what could be improved.

The trustworthiness of the findings was enhanced by the authors’ broad knowledge and experience in the field as nurses and researchers. Doing interviews in one’s own field may be challenging, since this can limit the scope of questions posed, and limits the exploration of topics one might take for granted. This might be why one of the interviews was very short and could be a limitation of the present study. Therefore, cautiousness is needed in the interpretation of the findings [36]. The authors were carefully analysing the data with this in mind, openly discussing our presuppositions, and the whole analysis process was discussed to minimize the risk of misinterpretations. We sought to ensure the transparency of the analytical approach by describing the method thoroughly and thereby increasing the credibility of the study. 

## 5. Conclusions

The purpose of the study was to gain an insight into what factors health professionals in home healthcare thought were important to provide good quality services and what could put safety at risk. An important factor expressed by the participants was that the nurses have sufficient competence, experience, and good training. Furthermore, quality could be linked to communication and information flow, where knowing the patient’s needs and communication between the various services around the patient was important for quality. A third factor concerned the way home healthcare was organized. Continuity and organization of tasks could affect the quality of care. Finally, quality and safety were linked to resources, such as adequate staffing and time to perform tasks in a good way. A deficiency within one or more of these factors, such as lack of training, low continuity, poor communication, or lack of time, could lead to poor quality and patient safety risks in the service of the individual patient. The findings are in line with previous research showing that several of these factors are important for good quality, and patient safety risks have been identified in several of the areas. Since this study revealed similar findings as in previous research, the question can be raised as to why it is so difficult to reduce the risk of quality deficiencies in the services. Recently, home healthcare quality has gained more attention. However, more investigation about why these known challenges are not being addressed, despite indications that this is highly needed, should be emphasized. There is a need to investigate in greater detail which factors influence quality and safety, and whether there are some conditions in home healthcare that contribute to these challenges.

## Figures and Tables

**Table 1:** Interview guide. Overview of themes and key questions.

Theme	Key Questions
Opening questions	What is your education and position now? What other units in healthcare do you have experience from? How long have you worked in home healthcare? What are the characteristics of the patients you care for now, and how many patients do you visit during a shift?
Quality of care	If I say good/bad quality of care in home healthcare, what are your immediate thoughts? What do you think about the quality of care in your unit? Are there situations or in cases you think negligence more easily happens? What do you think is the reason for carelessness or low quality of care in your unit?
What influences quality of care	What do you think is important for quality of care in home healthcare? What contribute to experiences of high quality of care in home healthcare? What needs to be changed for the quality to improve?

**Table 2:** Overview of main categories and subcategories.

A Workplace with Adequate Competence	Communication, Information Flow and Collaboration	Continuity and Organization of Care	Resources
Competence and experience Training and education	Considering the individual patient’s needs Communication between staff members Information and updating Interdisciplinary collaboration	Continuity in care Organization of tasks Work climate	Staffing/mix Time Reporting adverse events

## Data Availability

The datasets generated and/or analyzed during the current study are not publicly available due to format of the data not allowing for completely anonymizing data but are available from the corresponding author on reasonable request.
